# Identification of an *AR* Mutation-Negative Class of Androgen Insensitivity by Determining Endogenous AR Activity

**DOI:** 10.1210/jc.2016-1990

**Published:** 2016-09-01

**Authors:** N. C. Hornig, M. Ukat, H. U. Schweikert, O. Hiort, R. Werner, S. L. S. Drop, M. Cools, I. A. Hughes, L. Audi, S. F. Ahmed, J. Demiri, P. Rodens, L. Worch, G. Wehner, A. E. Kulle, D. Dunstheimer, E. Müller-Roßberg, T. Reinehr, A. T. Hadidi, A. K. Eckstein, C. van der Horst, C. Seif, R. Siebert, O. Ammerpohl, P.-M. Holterhus

**Affiliations:** Department of Pediatrics (N.C.H., M.U., J.D., P.R., A.E.K., P.-M.H.), Division of Pediatric Endocrinology and Diabetes, and Institute of Human Genetics (L.W., R.S., O.A.), Christian-Albrechts-University Kiel and University Hospital Schleswig-Holstein, Campus Kiel, Schwanenweg 20, 24105 Kiel, Germany; Rheinische Friedrich-Wilhelms-Universität Bonn, Department of Medicine III (H.U.S., G.W.), Institute for Biochemistry and Molecular Biology, Nussallee 11, 53115 Bonn, Germany; Department of Pediatrics (O.H., R.W.), Division of Experimental Pediatric Endocrinology, University of Luebeck, 23538 Luebeck, Germany; Department of Pediatrics (S.L.S.D.), Division of Pediatric Endocrinology, Sophia Childreńs Hospital, Erasmus Medical Center, 's-Gravendijkwal 230, 3015 CE Rotterdam, The Netherlands; Department of Pediatric Endocrinology (Medical Center), Ghent University Hospital, Ghent University, 9000 Ghent, Belgium; Department of Pediatrics (I.A.H.), University of Cambridge, Cambridge CB2 0QQ, United Kingdom; Pediatric Endocrinology Research Unit (L.A.), Vall d'Hebron Institut de Recerca, Hospital Universitari Vall d'Hebron, Centro de Investigación Biomédica en Red Enfermedades Raras, Instituto de Salud Carlos III, Passeig Vall d'Hebron 119, 08035 Barcelona, Spain; Developmental Endocrinology Research Group (S.F.A.), School of Medicine, University of Glasgow, Yorkhill Glasgow G3 8SJ, United Kingdom; Kinderklinik (D.D.), Klinikum Augsburg, 86156 Augsburg, Germany; Klinikum Esslingen (E.M.-R.), 73730 Esslingen, Germany; Department of Pediatrics (T.R.), Division of Pediatric Endocrinology, Diabetes, and Nutrition, University Witten/Herdecke, 45711 Datteln, Germany; Hypospadiezentrum (A.T.H.), 63500 Seligenstadt, Germany; Gemeinschaftspraxis für Kinderchirurgie (A.K.E.), 24119 Kronshagen, Germany; Urologische Gemeinschaftspraxis (C.v.d.H), and UROLOGIE Zentrum Kiel (C.S.), 24103 Kiel, Germany; and Institute of Human Genetics (R.S.), University of Ulm and University Hospital of Ulm, 89081 Ulm, Germany

## Abstract

**Context:**

Only approximately 85% of patients with a clinical diagnosis complete androgen insensitivity syndrome and less than 30% with partial androgen insensitivity syndrome can be explained by inactivating mutations in the androgen receptor (*AR*) gene.

**Objective:**

The objective of the study was to clarify this discrepancy by in vitro determination of AR transcriptional activity in individuals with disorders of sex development (DSD) and male controls.

**Design:**

Quantification of DHT-dependent transcriptional induction of the AR target gene apolipoprotein D (*APOD*) in cultured genital fibroblasts (GFs) (APOD assay) and next-generation sequencing of the complete coding and noncoding *AR* locus.

**Setting:**

The study was conducted at a university hospital endocrine research laboratory.

**Patients:**

GFs from 169 individuals were studied encompassing control males (n = 68), molecular defined DSD other than androgen insensitivity syndrome (AIS; n = 18), *AR* mutation-positive AIS (n = 37), and previously undiagnosed DSD including patients with a clinical suspicion of AIS (n = 46).

**Intervention(s):**

There were no interventions.

**Main Outcome Measure(s):**

DHT-dependent *APOD* expression in cultured GF and *AR* mutation status in 169 individuals was measured.

**Results:**

The APOD assay clearly separated control individuals (healthy males and molecular defined DSD patients other than AIS) from genetically proven AIS (cutoff < 2.3-fold *APOD*-induction; 100% sensitivity, 93.3% specificity, *P* < .0001). Of 46 DSD individuals with no *AR* mutation, 17 (37%) fell below the cutoff, indicating disrupted androgen signaling.

**Conclusions:**

*AR* mutation-positive AIS can be reliably identified by the APOD assay. Its combination with next-generation sequencing of the *AR* locus uncovered an *AR* mutation-negative, new class of androgen resistance, which we propose to name AIS type II. Our data support the existence of cellular components outside the *AR* affecting androgen signaling during sexual differentiation with high clinical relevance.

## Abstract (precis)

The use of Apolipoprotein D as a biomarker for androgen sensitivity identifies a new type of androgen insensitivity syndrome that is not associated with a mutation in the androgen receptor gene.

Sexual development is a complex process involving three crucial steps: development of the gonads in the embryo, synthesis of sex hormones, and sex hormone action. Genetic errors in any of these processes can lead to a wide range of sexual phenotypes that can be broadly included under the umbrella term of disorders of sex development (DSD) (1–3). Androgen insensitivity syndrome (AIS) (online inheritance in man number 300068) is a DSD that is classically characterized as a disorder of hormone action due to a reduced or absent functionality of the androgen receptor (AR) protein encoded by the *AR* gene. AIS is often suspected to be a common cause of DSD in a 46,XY individual and may be associated with complete feminization of the external genitalia due to a complete lack of AR transcriptional activity (complete AIS [CAIS]) (4), a variable level of feminization/masculinization due to a partial lack of transcriptional activity (partial AIS [PAIS]), or isolated male infertility (mild AIS [MAIS]).

For optimal function, the AR is activated through its ligands, T and the more potent DHT, after which it translocates into the nucleus and binds to its target genes whose expression entails the development of male internal and external genitalia. This process is tightly regulated through coactivators and corepressors of the AR (5, 6). Many AR target genes have been described in prostate cancer-derived cell lines; however, only a handful of genes have been identified in healthy male genital tissue (7). Among these, apolipoprotein D (*APOD*) has been reported to exhibit the most significant induction upon DHT treatment. *APOD* is a direct transcriptional target of the AR (8, 9), and a DHT-dependent secretion of APOD has been observed in prostate cancer cells (10). APOD belongs to the lipocalin protein family (11) and is able to carry *E*-3-methyl-2-hexenoic acid, the most abundant axillary odorant in males, to the skin surface ultimately used for pheromonal communication (12).

Whereas the clinical diagnosis of CAIS is relatively easy and can be confirmed by identifying a genetic abnormality in the *AR* coding sequence (*AR*-CDS) in more than 85% of cases, the clinical diagnosis of PAIS is more difficult, and, in addition, less than 30% of cases that are clinically suspected of PAIS are associated with a mutation in the *AR* (13). It is not known whether some individuals with 46,XY DSD may, in fact, have a currently unidentified new class of androgen insensitivity despite the absence of an *AR*-CDS mutation or whether some, or even all, rather have normal cellular AR function, thus excluding AIS.

To understand the possible coexistence of androgen resistance without any genetic evidence of a defect in the *AR*, we analyzed a cohort of 169 individuals including male controls, individuals with genetically proven AIS, and individuals with a clinical suspicion but no molecular proof of AIS in whom genital fibroblasts were available. Combining *AR*-sequencing analysis with a functional assay for AR activity by measuring the DHT-dependent transcriptional induction of the androgen-regulated *APOD* gene in cultured genital fibroblasts (GF) (APOD assay) enabled us to discover a new *AR* mutation-negative class of androgen resistance, which we propose to name AIS type II.

## Materials and Methods

The study was performed in agreement with the vote of the Ethical Committee of the Medical Faculty of the Christian-Albrechts-University (Kiel, Germany; AZ: D415/11; File S1). GFs received from collaborating partners were included in this study according to the recommendations of the local ethical committees. All GFs included in this study were double encrypted and numbered from 1 to 169.

### Sample collection

The GFs herein analyzed belonged to four major clinical groups.

**Figure 1.**
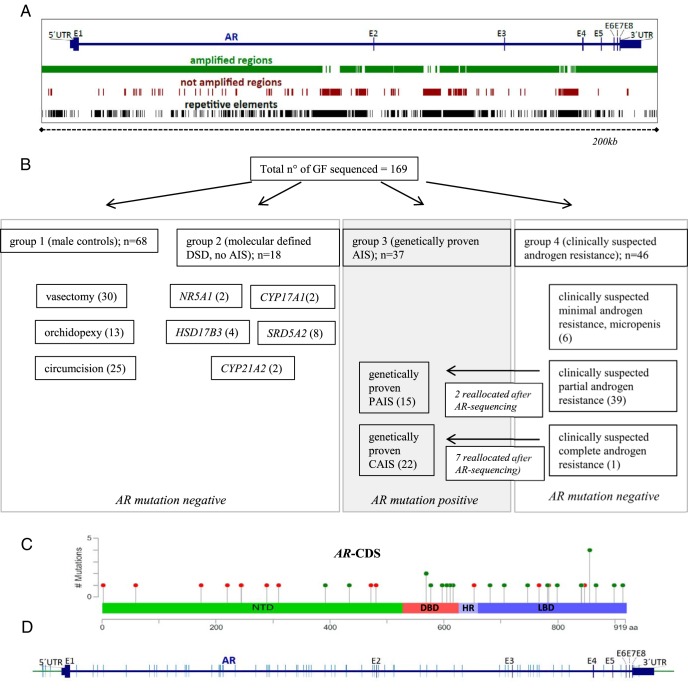
NGS of the *AR* locus. A, Graphic representation of the *AR* locus and the regions amplified by the haloplex design (chrX: 66,754,874–66955461 [hg19]) (shown in green). Highly repetitive sequences were excluded from the design (shown in brown). For comparison, repetitive elements present in this locus are shown in black. B, Division of the four patient groups from whom cultured GFs were analyzed. When mutations were found in the *AR*-CDS of GFs from group 4 (clinically suspected androgen resistance), they were reallocated to group 3 (genetically proven AIS). Therefore, two of the initially 41 samples from group 4 with clinically suspected PAIS and seven of the initially eight samples with clinically suspected CAIS were reallocated to group 3, resulting in 15 GF samples with genetically proven PAIS and 22 samples with genetically proven CAIS, respectively. C, Distribution of mutations found in the CDS of the *AR*. Red dots represent nonsense mutations and green dots missense mutations. Synonymous mutations in the coding region were not considered as CDS mutations. The graph was designed using the Mutation Mapper software from cBioPortal. D, Distribution of nonannotated SNPs along the sequenced region (green bars). DNTD, N-terminal transactivation domain; DBD, DNA-binding domain; HR, hinge region; LBD, ligand-binding domain.

The first group (group 1) was established in collaboration with local urologists and pediatric surgeons and includes scrotum-derived control GFs from fertile adult patients with normal virilization of the external genitalia, who underwent vasectomy (n = 30). We included scrotal biopsies of patients under the age of 18 years who underwent orchidopexy due to maldescended testes (n = 13) with normal external genitalia, ie, no hypospadias. In addition, we used control foreskin fibroblasts from patients who underwent circumcision due to cultural reasons or phimosis (n = 25). Genomic DNA of all male control GF cultures was sequenced using our custom haloplex next-generation sequencing (NGS) panel including up- and downstream sequences, untranslated regions, and the introns (Figure 1A).

The second group consists of GFs from previously characterized 46,XY DSD individuals with a defined molecular diagnosis other than AIS (group 2). In particular, these individuals carried mutations in the steroidogenic factor 1 gene (*NR5A1*) (n = 2), the 17α-hydroxylase gene (*CYP17A1*) (n = 2), the 17β-hydroxysteroid-dehydrogenase type III gene (*HSD17B3*) (n = 4), and the 5α-reductase type II gene (*SRD5A2*) (n = 8) in conjunction with ambiguous or female external genitalia. Biopsies were taken from either labioscrotal or foreskin/labia minora tissue. We added GFs from female (46,XX) individuals with congenital adrenal hyperplasia due to 21-hydroxylase deficiency (*CYP21A2*) (n = 2). Genomic DNA derived from the GF cultures of the second group was sequenced via the custom haloplex NGS *AR* panel.

The third group contains labioscrotal and foreskin/labia minora-derived GFs with a genetic proof of AIS, in whom mutations in the *AR*-CDS were either found previously via Sanger sequencing or in this paper through the haloplex NGS *AR* panel (n = 37; group 3). All GFs that revealed an *AR*-CDS mutation via the custom haloplex NGS *AR* panel were validated by Sanger sequencing.

The fourth group was compiled from a collection of labioscrotal and foreskin/labia minora-derived GF samples from 46,XY DSD individuals without an established molecular diagnosis (group 4). It includes individuals with apparently unaffected androgen biosynthesis based on available hormone data supporting a clinical suspicion of androgen resistance (n = 46). When available, data on the external genital appearance, basal and stimulated T levels (HCG test), and measurements on AR ligand binding (maximal binding capacity and dissociation constant) were collected (Supplemental Tables 1 and 2). This group may also contain individuals with a yet-undiagnosed form of DSD other than AIS. All GFs of group 4 were sequenced through our custom haloplex NGS *AR* panel. If an *AR*-CDS mutation was detected by NGS, Sanger sequencing was used for confirmation and the GFs were subsequently reallocated to group 3.

Supplemental Table 3 lists all GFs included in this study according to their location of biopsy together with the median age at biopsy.

Primary culturing of genital skin biopsies, the APOD assay, NGS library preparation, sequencing, and further methods are described in the Supplemental Material, including Supplemental Figures 1–7.

## Results

### Separation of GF into *AR* coding sequence mutation-positive and -negative entities

In the group of male controls (group 1), no mutations were detected in the coding sequence (CDS) and the intron-exon boundaries of the *AR*. In group 2 (molecular defined DSD diagnoses other than AIS), there were also no *AR*-CDS or intron-exon boundary mutations. In all classical AIS individuals (genetically proven AIS, group 3) previously identified by Sanger sequencing, mutations in the *AR*-CDS could be validated by our custom NGS *AR* panel, underlining the validity of the NGS approach. All those individuals in group 4 with previously undiagnosed forms of DSD in whom we identified a mutation in the *AR-*CDS by NGS (n = 9) were reallocated to group 3. In the remaining GF samples of group 4, including DSD samples in which AIS was suspected (n = 46), neither mutations in the *AR-*CDS nor in the intron-exon boundaries could be detected. A schematic representation of all four groups is shown in Figure 1B. The distribution of detected *AR*-CDS mutations within group 3 is schematically shown in Figure 1C, and their exact position is listed in Supplemental Table 4. Eight *AR-CDS* mutations are not currently listed in the AR mutation database (14) and, to our knowledge, have not been described in the literature. These unreported mutations are frameshift mutations (n = 5), stop mutations (n = 1), and missense mutations (n = 2) (Supplemental Table 4). Outside the coding region, numerous nonannotated single-nucleotide polymorphisms (SNPs) were found in all four groups. A distribution of those SNPs is shown in Figure 1D.

### Calculation of a cutoff for the functional classification of male controls (group 1), molecular-defined DSD other than AIS (group 2), and *AR*-CDS mutation-positive AIS individuals (group 3) using the APOD assay

**Figure 2.**
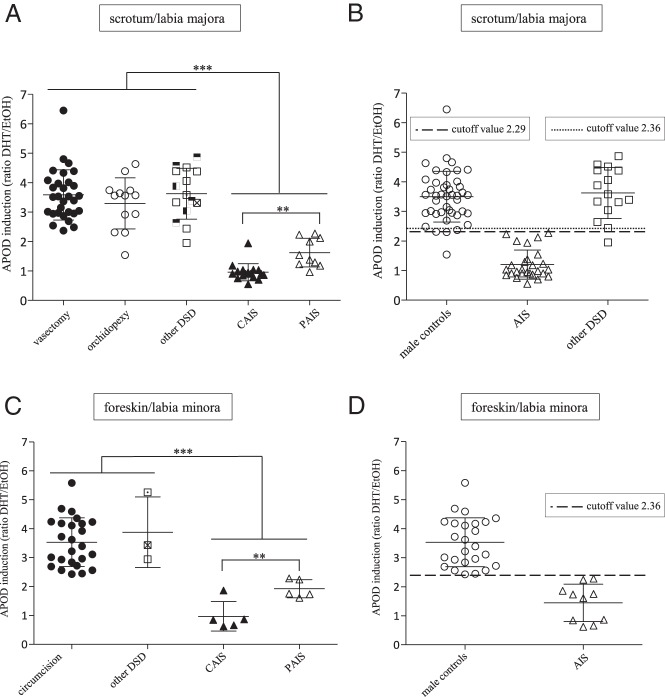
DHT-dependent, AR-induced *APOD* mRNA expression represented as the ratio between ethanol (EtOH)- and DHT-treated GFs. A, Scrotum-derived male controls (vasectomy, orchidopexy [group 1]), labioscrotal derived molecular defined DSDs (other DSDs [group 2]), and *AR*-CDS mutation-positive AIS (CAIS, PAIS [group 3]). B, Depiction of cutoff values between male controls (vasectomy and orchidopexy) and *AR*-CDS mutation-positive AIS (CAIS and PAIS) of 2.29 (100% sensitivity, 97.7% specificity, *P* < .0001) and between the same *AR*-CDS mutation-positive AIS and molecular defined DSDs (other DSDs) of 2.36 (100% sensitivity, 93,3% specificity, *P* < .0001). C, Foreskin-derived male controls (circumcision [group 1]) and molecular-defined DSDs (other DSDs [group 2[) as well as *AR*-CDS mutation-positive AIS (CAIS, PAIS [group 3]). D, Depiction of the cutoff value between male controls (circumcision) and *AR*-CDS mutation-positive AIS (CAIS and PAIS) of 2.36 (100% sensitivity, 100% specificity, *P* < .0001). Means and SDs are included as error bars. Values of *P* < .001 are denoted by three stars, and those values of *P* < .01 are denoted by two stars. Among the DSD diagnoses other than AIS, empty squares represent *SRD5A2*, horizontally half-filled squares represent *HSD17B3*, vertically half-filled squares represent *CYP17A1*, crossed squares represent *CYP21A2*, and dotted squares represent *NR5A1* mutations.

We now functionally characterized all 169 sequenced GF by measuring the DHT-triggered ability of the AR to induce transcription of its target gene *APOD* (APOD assay). Male control scrotum-derived GFs from group 1 (vasectomy, orchidopexy) showed a mean DHT-mediated *APOD* induction of 3.5-fold (SD 0.85), defining the normal range of transcriptional function of the AR in this group (Figure 2A). Scrotum-derived GFs from group 2 (molecular defined DSD other than AIS) showed the same degree of *APOD* up-regulation, confirming uncompromised functionality of the AR in these cells (Figure 2A). Only one orchidopexy-derived control GF cell line from an individual in group 1 showed an unexpectedly low induction of *APOD*. In the light of the complete data set provided in this manuscript and because the final steps of the testicular descent are androgen dependent, we retrospectively have to conclude that this individual has some degree of androgen resistance (15). In contrast to groups 1 and 2, *APOD* induction was significantly lower in scrotum-derived GFs from classical AIS individuals (group 3) (*P* < .001). CAIS-derived GFs showed on average no induction (0.96), whereas PAIS-derived GFs demonstrated an average induction of 1.62 (Figure 2A). This confirms androgen resistance at the functional molecular level in these cells and underlines the validity of the APOD assay. We now calculated the cutoff between scrotal-derived control GFs from group 1 (adults and children together) and the corresponding labioscrotal-derived GFs from AIS individuals in group 3 (CAIS and PAIS together). Consequently, an *APOD* induction below 2.29 represents a form of androgen resistance with a sensitivity of 100% and a specificity of 97.67% and indicates that the two groups are separable with high confidence (Figure 2, A and B, and Supplemental Figure 8A).

From the clinical perspective, it is of much greater relevance to distinguish AIS from other forms of DSD rather than from clinically unsuspicious male controls. We calculated a cutoff between labioscrotal-derived GFs from group 2 (molecular defined DSD other than AIS) and group 3 (*AR*-CDS mutation positive AIS) of 2.36-fold *APOD* induction. Hence, a DHT-mediated *APOD* induction less than 2.36 distinguishes genetically proven AIS from other molecular-defined DSDs with a sensitivity of 100% and a specificity of 93.33% (Figure 2, A and B, and Supplemental Figure 8B).

When analyzing foreskin/labia minora-derived GFs, the APOD assay could again reliably separate male control fibroblasts (group 1) and GFs from AIS individuals harboring an *AR*-CDS mutation (group 3) (Figure 2C). A cutoff of 2.36-fold *APOD* induction was determined for foreskin/labia minora-derived tissue with 100% sensitivity and specificity (Figure 2, C and D, and Supplemental Figure 8C). The average DHT-mediated *APOD* induction in CAIS was 0.97 (no *APOD* induction) and 1.92 in PAIS (Figure 2B). Although GFs from the individuals of group 2 showed an as high *APOD* induction as male controls (group 1) (Figure 2C), no cutoff could be calculated because there were not enough corresponding foreskin-derived GF strains available in our DSD-GF biobank. When testing two GF strains derived from congenital adrenal hyperplasia individuals carrying *CYP21A2* mutations and having a 46,XX karyotype, their *APOD* response to DHT was comparable with that of male controls, confirming that the AR can be activated by DHT in GF independently of the chromosomal sex (Figure 2, A and B).

**Figure 3.**
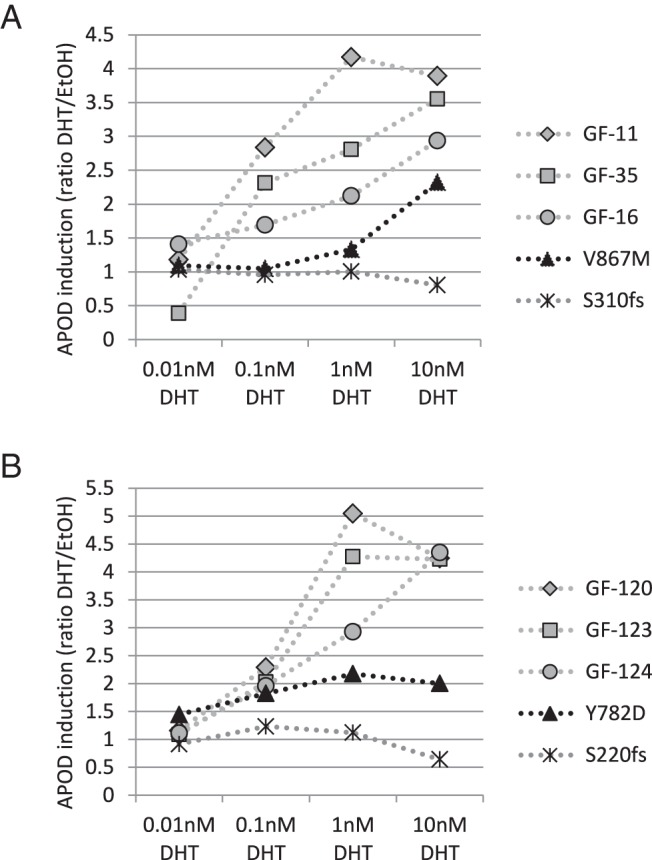
DHT-dependent, AR-induced *APOD* induction in response to different DHT concentrations in the culture media. A, GF-11, GF-16, and GF-35 are scrotum-derived male control GFs (Supplemental Table 1). GFs derived from a CAIS patient carrying the p.Ser310fs mutation served as negative control. The p.Val867Met mutation is shown in black. B, GF-120, GF-123, and GF-124 are foreskin-derived male control GFs (Supplemental Table 2). GFs derived from a CAIS patient carrying the p.Ser220fs mutation served as the negative control. The p.Tyr782Asp mutation is shown in black.

We than examined whether GFs derived from genetically proven CAIS and PAIS individuals within group 3 could be distinguished from each other by the APOD assay. When comparing DHT-mediated *APOD* induction, we found a significant difference between PAIS and CAIS in both labioscrotal and labia minora/foreskin-derived tissues (*P* < .01). However, there was some overlap due to a few GF cultures (Figure 2, A and C). One GF cell line carrying a p.Val867Met mutation in the ligand binding domain of the AR derived from a CAIS individual still showed residual *APOD* induction. Interestingly, when using lower DHT concentrations, *APOD* induction was abolished (Figure 3A). Labia minora-derived GFs from another CAIS individual bearing the mutation p.Tyr782Asp, again located in the AR-ligand binding domain, also showed residual *APOD* induction. However, this partial activity was even present at lower DHT concentrations (Figure 3B). A third GF cell line was derived from an individual with predominantly female external genitalia and therefore PAIS, carrying a p.Leu174stop mosaic. The latter was present in 94% of the cultured GFs according to the NGS reads, which is most likely the cause for complete abolishment of DHT-mediated *APOD* induction despite the PAIS phenotype.

We also compared *APOD* induction between GFs harboring nonsense or missense mutations in the AR protein. Nonsense mutations (stop or frame shift mutations) never showed any *APOD* induction and always belonged to the CAIS group (apart from the p.Leu174stop mosaic), whereas missense mutations had a variable *APOD* induction and were present in both PAIS- and CAIS-derived GFs (see Supplemental Tables 1 and 2).

In conclusion, the APOD assay does distinguish CAIS individuals from PAIS individuals, albeit with slightly lower sensitivity (88.2%) and specificity (90%) (Supplemental Figure 8D).

### AR activity in *AR*-CDS mutation-negative GFs from individuals with suspected diagnosis of AIS (group 4)

**Figure 4.**
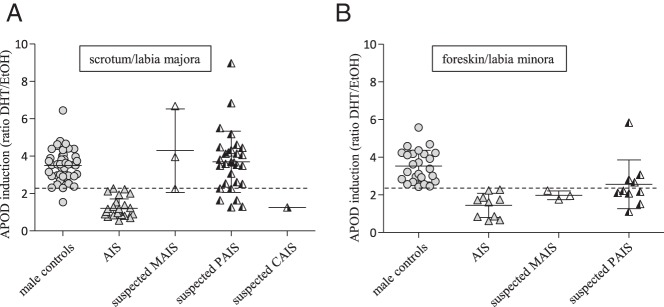
DHT-dependent, AR-induced *APOD* expression in *AR*-CDS-negative individuals with clinically suspected androgen resistance (group 4) derived from scrotum/labia majora (A) and from foreskin/labia minora (B). Suspected androgen-resistant GFs of group 4 are divided into mild androgen resistance (MAIS, micropenis), partial androgen resistance (PAIS, ambiguous external genitalia), and complete androgen resistance (CAIS, completely female external genitalia). Included are means and SDs. For comparison, the tissue-specific controls and *AR*-CDS mutation-positive GFs are shown as well. The calculated cutoffs are drawn as dotted lines.

We then analyzed the large group of *AR*-CDS mutation-negative GFs derived from individuals with no previously established DSD diagnosis (group 4) using the APOD assay and applied the above calculated cutoffs. Looking at labioscrotal-derived GFs, 24% (n = 8) of fibroblast cultures from group 4 fell below the cutoff of 2.29 and therefore have to be defined as functionally androgen resistant (Figure 4A and Supplemental Table 1). One of the GF cultures was from an individual with the suspected clinical diagnosis of CAIS and showed strongly reduced *APOD* induction. In contrast, the remaining 76% (n = 25) GF cell lines showed an *APOD* induction above the cutoff and had an AR activity comparable with that of control groups 1 and 2 (Figure 4A). Therefore, these GF cell lines have to be defined as normally androgen responsive. Analyzing the foreskin/labia minora-derived GFs in group 4, the majority (69%, n = 9) fell below the cutoff of 2.36. Again, these cultures have to be defined as androgen insensitive on a functional basis (Figure 4B and Supplemental Table 2). The remaining 31% of foreskin/labia minora GF cultures (n = 4) behaved like male foreskin controls (group 1) in terms of *APOD* induction, and hence, androgen insensitivity can be ruled out. In summary, 17 of the 46 GFs from group 4 have functionally proven androgen resistance based on androgen-induced *APOD* transcription despite the absence of an *AR*-CDS mutation.

### Molecular characterization of the *AR*-CDS mutation-negative but functionally androgen-resistant GFs

**Figure 5.**
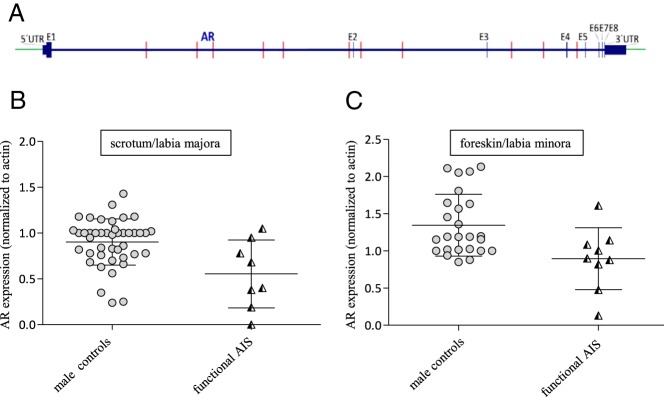
Analysis of *AR*-CDS mutation-negative but functionally androgen-insensitive GF. A, Distribution of potentially damaging mutations in the sequenced region outside the *AR*-CDS. B, AR protein expression in male scrotum-derived controls and *AR*-CDS mutation-negative but functionally androgen-insensitive labioscrotal GFs. C, AR protein expression in male foreskin-derived controls and *AR*-CDS mutation-negative but functionally androgen-insensitive GFs. Included are means and SDs.

Finally, we wanted to know whether mutations detected within the *AR* locus but outside the *AR*-CDS in individuals of group 4 could potentially have influenced AR activity. Of the 17 *AR*-CDS-mutation-negative, androgen-resistant GFs, nine had one or more not annotated SNPs outside the *AR*-CDS, whereas eight had only previously annotated and clinically unsuspicious SNPs in the region covered by our NGS approach. The distribution of the nonannotated SNPs along the analyzed *AR* locus is shown in Figure 5A. We speculated that a low *APOD* induction could be due to a reduced AR protein expression or stability in these GFs. We checked the AR protein levels in all the 17 GF cultures and compared them with their appropriate control groups (scrotal and foreskin derived GFs) (Figure 5, B and C, and Table 1). A lower AR protein expression was seen in four GF of the *AR*-mutation-negative, androgen-resistant group, indicating that AR protein expression was impaired in these cases. Two of these four individuals had nonannotated SNPs outside the CDS (Table 1). In conclusion, we show that reduced AR protein expression or stability can explain a reduced *APOD* induction in about one-fourth of the analyzed cases.

**Table 1.** List of *AR*-CDS Mutation-Negative but Functionally Androgen-Insensitive GFs

GF	Origin of Biopsy	SNP (Chromosome Reference>Alternate)	APOD Induction	AR Protein Expression	Encode Regulation (hg19)
GF-89	S	Nothing	1.26	0.00	
GF-107	S	ChrX:66912572 G>A	2.27	0.19	Strong enhancer in HSMM
GF-104	S	Nothing	1.30	0.38	
GF-105	S	Nothing	1.64	0.40	
GF-90	S	ChrX:66839548 G>A	2.26	0.68	Strong enhancer in HUVEC
GF-109	S	ChrX:66811878 T>C	1.64	0.78	Polycomb repressed in GM12878, K562, H1-hESC, HELA, HUVEC, HepG2
GF-86	S	Nothing	2.25	0.95	
GF-118	S	ChrX:66922786 A>G	1.25	1.05	/
GF-164	F	ChrX:66877648 G>A	1.52	0.13	Polycomb repressed in GM12878, K562, H1-hESC, HELA, HUVEC, HepG2
GF-158	F	Nothing	1.76	0.48	
GF-162	F	ChrX:66864354 A>G; ChrX:66860551 C>A	2.08	0.82	Polycomb repressed in GM12878, K562, H1-hESC, HELA, HUVEC, HepG2
GF-160	F	ChrX:66795584 A>G; ChrX:66817032 3bp del	2.11	0.88	Polycomb repressed in GM12878, K562, H1-hESC, HELA, HUVEC, HepG2; Pol2-associated transcription in H1-hESC
GF-159	F	ChrX:66933579 G>C	1.95	0.90	Transcription in HUVEC
GF-168	F	Nothing	2.17	1.00	
GF-157	F	Nothing	2.23	1.09	
GF-163	F	ChrX:66833033 A>G	2.22	1.14	Strong enhancer in HUVEC and HSMM
GF-166	F	Nothing	1.11	1.61	

Abbreviations: GM12878, B-lymphocyte; HELA, cervical carcinoma cells; ; HepG2, liver hepatocellular carcinoma cells H1-hESC, H1 human embryonic stem cells; HSMM, human skeletal muscle cells and myoblasts; HUVEC, human umbilical vein endothelial cells; K562, leukemia cell line; S, scrotal derived GF; F, foreskin derived GF.

## Discussion

Functional assays for AR activity in GFs have been described before (16). Lacking a target gene for the AR, however, they were dependent on the transfection of exogenous reporter constructs to monitor endogenous AR activity. We here provide the APOD assay as a tool for the functional characterization of cellular AR function in GFs derived from DSD patients. We validate its diagnostic suitability in a very large cohort using male controls and various molecular well-defined DSD patients other than AIS as well as several genetically proven classical AIS individuals. The resulting diagnostic cutoffs not only helped to exclude the diagnosis of AIS in many cases but also lead to the identification of an androgen-resistant but *AR*-CDS-negative new class of AIS, which we suggest to call AIS type II. This is not only a significant addition to the current classification of 46,XY DSDs but also a starting point for a better understanding of AR signaling, including the identification of new AR cofactors in future clinical and molecular DSD studies.

Whereas the APOD assay separates classical AIS from male controls with high sensitivity and specificity, the separation was slightly less specific when comparing classical AIS from defined DSD diagnoses other than AIS. In fact, one individual with documented 5α-reductase (5αRD) deficiency (group2) showed reduced AR activity in the APOD assay. A possible explanation could be that this individual has a defect both in DHT synthesis and in androgen action. An additional 5αRD deficiency may also be responsible for the CAIS phenotype of an individual carrying a p.Tyr781Asp mutation in the ligand binding domain of the AR because biochemical data indicate reduced 5αRD activity in the GFs of this patient (identification in C31 [17]). Both the residual *APOD* in induction shown in this paper and in previously published DHT binding and dissociation studies (17) indicate only an incomplete loss of AR function in this individual despite a complete female phenotype.

Also, the APOD assay did not distinguish between PAIS and CAIS in all cases. This overlap may be affected by specific functional and molecular conditions in some individual GF cultures. In GFs from a CAIS individual carrying a p.Val867Met mutation in the AR ligand binding domain, we observed residual *APOD* induction with 10 nM DHT despite a clinical CAIS. Interestingly, no *APOD* induction was measured when using 1 nM DHT, suggesting that the CAIS phenotype might have originated from low local genital DHT concentrations during embryogenesis. This observation is supported by the literature associating this mutation with different AIS phenotypes, ranging from CAIS through PAIS to MAIS (14). Another phenomenon with functional relevance for the APOD assay may be the presence of somatic mosaicism, which is an apparently frequent condition in AIS due to the high new mutation rate (18, 19). In the PAIS subgroup within group 3 of our study, one cell line contained a p.Leu174stop mosaic (20) present in 94% of the cultured GFs according to the NGS reads. No *APOD* induction could be detected, well in line with the high percentage of the mutation in this PAIS cell culture. Hence, whereas the APOD assay correctly identified AIS in this situation, somatic mosaicism may influence the detected level of functional impairment, which may be in contrast to the clinical phenotype. We have previously described this phenomenon of discrepancy between molecular studies and the clinical phenotype in mosaic AIS, using other functional approaches (19, 21). Ultimately, due to the limited information regarding the exact AIS grades of the genital phenotypes in our DSD-GF biobank (eg, according to Quigley et al [22] or to Ahmed et al [23]), we cannot provide a meaningful AIS grade/APOD assay correlation to date. The APOD assay is therefore currently not a statistically proven tool for assessing the quantitative extent of androgen resistance in a given individual with DSD.

By analyzing sequencing data of the *AR* locus outside the *AR*-CDS, we could detect as-yet nonannotated SNPs within potentially regulatory regions. Some of these SNPs are potential candidates for influencing AR activity because they are paralleled by reduced AR protein expression in the corresponding GF cultures, which could explain the lower AR activity in the AIS type II individuals. Interestingly, we previously detected a mutation in the 5′untranslated region of the *AR* in an individual having CAIS and experimentally showed that this mutation is sufficient to strongly reduce AR protein levels and AR activity (24). This underlines the importance of detection of potential mutations outside the *AR*-CDS. Another promising group of factors outside the *AR* gene region that might contribute to AIS type II are AR cofactors, which are needed for proper AR activity (5, 6). Numerous cofactors of the AR have been described in prostate cancer (25), but a coregulator that exclusively regulates the AR has not been described so far. Since the *AR* gene was cloned in 1988 (26, 27), only one single case of disrupted AR activation through a coactivator defect has been reported in a CAIS individual (28), but this coactivator has never been identified. No further case has since been described.

We do not yet know whether the AIS type II cohort identified in this study has a monogenic origin or whether multiple aberrant genes may contribute to this entity. Exome sequencing of the *AR*-CDS-negative AIS type II cohort in comparison with the other three cohorts of this study is one of the next important experimental steps planned. Furthermore, we cannot exclude that mild functional AIS type II may play a role as secondary modifier contributing to a DSD phenotype, even in certain molecular-defined DSDs and in unknown DSDs. This is supported by previous reports documenting the existence of more than one compromised molecular factor in the same DSD individual (29–31). According to Cox et al (32), associated conditions occur in about a quarter of analyzed DSD cases. Looking specifically at cases with suspected androgen insensitivity syndrome, 11% anomalies were reported. In our *AR*-CDS-negative in AIS type II cohort, we found documented minor syndromic signs in 4 of 46 cases, hence 9%. In addition, prenatal conditions leading to low birth weight may have programming effects on androgen responsiveness of genital cells as a correlation of a low birth weight and a PAIS-like phenotype in individuals without an *AR* gene mutation has been described before (33).

Currently our data are based on retrospective analyses of fibroblasts obtained from our DSD biobank, but they can nevertheless be of potential value for the clinical endocrinologist. Apart from being an explanation for the phenotypic development of a DSD individual, reduced *APOD* induction may be associated with a reduced future AR sensitivity during puberty and may influence clinical response to androgen treatment. Prospective data are needed to correlate *APOD* expression with clinical outcome parameters in affected individuals. Given the high significance of the data provided in this manuscript, the scientific community in DSD research should revisit the clinical indication of a diagnostic genital skin biopsy in specific unclear DSD cases.

## References

[1] Arboleda VA Sandberg DE Vilain E DSDs: genetics, underlying pathologies and psychosexual differentiation. Nat Rev Endocrinol. 2014;10:603–615. 25091731 10.1038/nrendo.2014.130 PMC4441533

[2] Hiort O Birnbaum W Marshall L Management of disorders of sex development. Nat Rev Endocrinol. 2014;10:520–529. 25022812 10.1038/nrendo.2014.108

[3] Hiort O Ahmed SF Understanding differences and disorders of sex development. Endocr Dev. 2014;27:VII–VIII. 25373247 10.1159/isbn.978-3-318-02559-0

[4] Mongan NP Tadokoro-Cuccaro R Bunch T Hughes IA Androgen insensitivity syndrome. Best Pract Res Clin Endocrinol Metab. 2015;29:569–580. 26303084 10.1016/j.beem.2015.04.005

[5] van de Wijngaart DJ Dubbink HJ van Royen ME Trapman J Jenster G Androgen receptor coregulators: recruitment via the coactivator binding groove. Mol Cell Endocrinol. 2012;352:57–69. 21871527 10.1016/j.mce.2011.08.007

[6] Heemers HV Tindall DJ Androgen receptor (AR) coregulators: a diversity of functions converging on and regulating the AR transcriptional complex. Endocr Rev. 2007;28:778–808. 17940184 10.1210/er.2007-0019

[7] Appari M Werner R Wunsch L Cario G Apolipoprotein D (APOD) is a putative biomarker of androgen receptor function in androgen insensitivity syndrome. J Mol Med (Berl). 2009;87:623–632. 19330472 10.1007/s00109-009-0462-3 PMC5518750

[8] Tan PY Chang CW Chng KR Wansa KD Sung WK Cheung E Integration of regulatory networks by NKX3–1 promotes androgen-dependent prostate cancer survival. Mol Cell Biol. 2012;32:399–414. 22083957 10.1128/MCB.05958-11 PMC3255774

[9] Chng KR Chang CW Tan SK A transcriptional repressor co-regulatory network governing androgen response in prostate cancers. EMBO J. 2012;31:2810–2823. 22531786 10.1038/emboj.2012.112 PMC3380210

[10] Simard J Veilleux R de Launoit Y Haagensen DE Labrie F Stimulation of apolipoprotein D secretion by steroids coincides with inhibition of cell proliferation in human LNCaP prostate cancer cells. Cancer Res. 1991;51:4336–4341. 1868457

[11] Flower DR Beyond the superfamily: the lipocalin receptors. Biochim Biophys Acta. 2000;1482:327–336. 11058773 10.1016/s0167-4838(00)00169-2

[12] Zeng C Spielman AI Vowels BR Leyden JJ Biemann K Preti G A human axillary odorant is carried by apolipoprotein D. Proc Natl Acad Sci USA. 1996;93:6626–6630. 8692868 10.1073/pnas.93.13.6626 PMC39076

[13] Ahmed SF Bashamboo A Lucas-Herald A McElreavey K Understanding the genetic aetiology in patients with XY DSD. Br Med Bull. 2013;106:67–89. 23529942 10.1093/bmb/ldt008

[14] Gottlieb B Beitel LK Nadarajah A Paliouras M Trifiro M The androgen receptor gene mutations database: 2012 update. Hum Mutat. 2012;33:887–894. 22334387 10.1002/humu.22046

[15] Virtanen HE Toppari J Embryology and physiology of testicular development and descent. Pediatr Endocrinol Rev. 2014;11(suppl 2):206–213. 24683945

[16] McPhaul MJ Schweikert HU Allman DR Assessment of androgen receptor function in genital skin fibroblasts using a recombinant adenovirus to deliver an androgen-responsive reporter gene. J Clin Endocrinol Metab. 1997;82:1944–1948. 9177411 10.1210/jcem.82.6.3966

[17] Audi L Fernandez-Cancio M Carrascosa A Novel (60%) and recurrent (40%) androgen receptor gene mutations in a series of 59 patients with a 46,XY disorder of sex development. J Clin Endocrinol Metab. 2010;95:1876–1888. 20150575 10.1210/jc.2009-2146

[18] Holterhus PM Wiebel J Sinnecker GHG Clinical and molecular spectrum of somatic mosaicism in androgen insensitivity syndrome. Pediatr Res. 1999;46:684–690. 10590024 10.1203/00006450-199912000-00009

[19] Hiort O Sinnecker GH Holterhus PM Nitsche EM Kruse K Inherited and de novo androgen receptor gene mutations: investigation of single-case families. J Pediatr. 1998;132:939–943. 9627582 10.1016/s0022-3476(98)70387-7

[20] Holterhus PM Bruggenwirth HT Hiort O Mosaicism due to a somatic mutation of the androgen receptor gene determines phenotype in androgen insensitivity syndrome. J Clin Endocrinol Metab. 1997;82:3584–3589. 9360511 10.1210/jcem.82.11.4375

[21] Hiort O Sinnecker GH Holterhus PM Nitsche EM Kruse K The clinical and molecular spectrum of androgen insensitivity syndromes. Am J Med Genet. 1996;63:218–222. 8723113 10.1002/(SICI)1096-8628(19960503)63:1<218::AID-AJMG38>3.0.CO;2-P

[22] Quigley CA De Bellis A Marschke KB el-Awady MK Wilson EM French FS Androgen receptor defects: historical, clinical, and molecular perspectives. Endocr Rev. 1995;16:271–321. 7671849 10.1210/edrv-16-3-271

[23] Ahmed SF Khwaja O Hughes IA The role of a clinical score in the assessment of ambiguous genitalia. BJU Int. 2000;85:120–124. 10619959 10.1046/j.1464-410x.2000.00354.x

[24] Hornig NC de Beaufort C Denzer F A recurrent germline mutation in the 5′UTR of the androgen receptor causes complete androgen insensitivity by activating aberrant uORF translation. PloS One. 2016;11:e0154158. 27110943 10.1371/journal.pone.0154158 PMC4844194

[25] Culig Z Santer FR Molecular aspects of androgenic signaling and possible targets for therapeutic intervention in prostate cancer. Steroids. 2013;78:851–859. 23643785 10.1016/j.steroids.2013.04.012

[26] Chang CS Kokontis J Liao ST Molecular cloning of human and rat complementary DNA encoding androgen receptors. Science. 1988;240:324–326. 3353726 10.1126/science.3353726

[27] Trapman J Klaassen P Kuiper GG Cloning, structure and expression of a cDNA encoding the human androgen receptor. Biochem Biophys Res Commun. 1988;153:241–248. 3377788 10.1016/s0006-291x(88)81214-2

[28] Adachi M Takayanagi R Tomura A Androgen-insensitivity syndrome as a possible coactivator disease. New Engl J Med. 2000;343:856–862. 10995865 10.1056/NEJM200009213431205

[29] Hersmus R van der Zwan YG Stoop H A 46,XY female DSD patient with bilateral gonadoblastoma, a novel SRY missense mutation combined with a WT1 KTS splice-site mutation. PloS One. 2012;7:e40858. 22815844 10.1371/journal.pone.0040858 PMC3399878

[30] Idkowiak J Malunowicz EM Dhir V Concomitant mutations in the P450 oxidoreductase and androgen receptor genes presenting with 46,XY disordered sex development and androgenization at adrenarche. J Clin Endocrinol Metab. 2010;95:3418–3427. 20410220 10.1210/jc.2010-0058 PMC3071629

[31] Boehmer AL Brinkmann AO Nijman RM Phenotypic variation in a family with partial androgen insensitivity syndrome explained by differences in 5α dihydrotestosterone availability. J Clin Endocrinol Metab. 2001;86:1240–1246. 11238515 10.1210/jcem.86.3.7333

[32] Cox K Bryce J Jiang J Novel associations in disorders of sex development: findings from the I-DSD Registry. J Clin Endocrinol Metab. 2014;99:E348–E355. 24302751 10.1210/jc.2013-2918 PMC3955252

[33] Lek N Miles H Bunch T Low frequency of androgen receptor gene mutations in 46 XY DSD, and fetal growth restriction. Arch Dis Childh. 2014;99:358–361. 24366239 10.1136/archdischild-2013-305338

